# Differential effect of surgical manipulation on gene expression in normal breast tissue and breast tumor tissue

**DOI:** 10.1186/s10020-018-0058-x

**Published:** 2018-11-16

**Authors:** Inge Søkilde Pedersen, Mads Thomassen, Qihua Tan, Torben Kruse, Ole Thorlacius-Ussing, Jens Peter Garne, Henrik Bygum Krarup

**Affiliations:** 1 Molecular Diagnostics, Clinical Biochemistry, Aalborg University, Aalborg, Denmark; 2 Department of Clinical Genetics, Odense University Hospital, Odense, Denmark; 3 Department of Gastrointestinal Surgery, Aalborg University Hospital, Aalborg, Denmark; 4 Department of Breast Surgery, Aalborg University Hospital, Aalborg, Denmark; 5 Clinical Cancer Research Center, Aalborg University Hospital, Aalborg, Denmark; 6 Department of Clinical Medicine, Aalborg University, Aalborg, Denmark

**Keywords:** Specimen handling, Ischemia, Gene expression, Cell cycle, Cancer

## Abstract

**Background:**

Gene expression profiles of normal and tumor tissue reflect both differences in biological processes taking place in vivo and differences in response to stress during surgery and sample handling. The effect of cold (room temperature) ischemia in the time interval between surgical removal of the specimen and freezing is described in a few studies. However, not much is known about the effect of warm (body temperature) ischemia during surgery.

**Methods:**

Three women with primary operable breast cancer underwent in situ biopsies from normal breast and tumor tissue prior to radical mastectomy. Ex vivo biopsies from normal and tumor tissue were collected immediately after surgical excision. The putative effects on gene expression of malignancy (tumor versus normal), surgical manipulation (post- versus pre-surgical) and interaction between the two (differences in effect of surgical manipulation on tumor and normal samples) were investigated simultaneously by Generalized Estimating Equation (GEE) analysis in this self-matched study.

**Results:**

Gene set enrichment analysis (GSEA) demonstrates a marked difference in effect of surgical manipulation on tumor compared to normal tissue. Interestingly, a large proportion of pathways affected by ischemia especially in tumor tissue are pathways considered to be specifically up regulated in tumor tissue compared to normal.

**Conclusion:**

The results of this study suggest that a large contribution to this differential expression originates from altered response to stress in tumor cells rather than merely representing in vivo differences. It is important to bear this in mind when using gene-expression analysis to deduce biological function, and when collecting material for gene expression profiling.

**Electronic supplementary material:**

The online version of this article (10.1186/s10020-018-0058-x) contains supplementary material, which is available to authorized users.

## Introduction

Several multigene classifiers are currently commercially available for breast cancer, among them MammaPrint (Agendia, The Netherlands), Oncotype DX (Genomic Health, USA) and PAM50 (Nano String, USA). However, caution should be taken interpreting gene signatures, when sampling conditions are suboptimal (i.e. prolonged delay of sample preservation after surgery), since sample handling and preservation methods affect gene expression. This has been shown previously by snap freezing aliquots of breast tumor tissue at different time points after surgical removal in a few studies. De Cecco et al. (2009) investigated breast tumor tissue from 11 patients. For each patient 4 aliquots were analyzed, 1 was frozen immediately and the remaining left at room temperature for 2, 6 and 24 h respectively. No major effect on RNA integrity was observed. However, expression levels of 461 genes were significantly altered. Borgan et al. (2011) observed significantly altered expression of 1788 mRNAs and 56 miRNAs in a study with delayed freezing of 0.5, 1, 2, 3 and 6 h when compared to the breast tumor tissue frozen immediately post surgery. Aktas et al. (2014) investigated the difference between preservation of breast tumor tissue in RNAlater and freezing on dry ice in prechilled vials, they found expression of 481 transcripts to be significantly altered. In addition, they observed an effect of delayed preservation on 41 transcripts. In a previous study on the same samples, comparing preservation methods, it was concluded that RNAlater improves RNA yield and quality (Hatzis et al., 2010). Recently the sparse literature on the effect of cold ischemia has been reviewed (Grizzle et al., 2016).

As pointed out by Grizzle et al., warm ischemia may be the more important variable, however this is not easily controlled and data on the effect of warm ischemia is rarely available (Grizzle et al., 2016) . To our knowledge no study has investigated the effect of surgical manipulation on gene expression profiles in breast tissue. In animal studies a difference between effect of ischemic stress at room temperature and body temperature has been shown (Almeida et al., 2004). In addition, a study investigating gene expression in histologically benign prostate tissue before and after surgery demonstrated substantial gene expression changes as a result of surgical manipulation with a median warm ischemia time of 28 min during surgery (Lin et al., 2006). Albeit, the effect is likely to be at least partly contributable to ischemia, other factors may also affect gene expression. The best studied example being anesthesia (Lucchinetti et al., 2007). Since environmental changes cause the cell to adjust its regulatory mechanisms which in turn alters the way it reacts to external input, tumor and normal cells are not only different per se, they could also be expected to react differently when exposed to stress such as surgical manipulation. The current study has been designed to investigate the effect of surgical manipulation in both normal breast tissue and breast tumor tissue by comparing gene expression profiles before and after surgery using a self-matched study design for increased statistical power.

## Materials and methods

### Patient material

Participants were 3 women with primary operable breast cancer. None of the participants had received anti-cancer treatment prior to surgery. Time between biopsy was approximately 1 h in the 3 cases. Clinical and pathological characteristics of the patients are summarized in Table 1. The study protocol was approved by the Research Ethics Committee of the North Denmark Region (N-20090029). All participants gave written informed consent.

**Table 1:** Patient cilincal ad pathological charcteristics and details of sample collection

Patient ID	Age at diagnosis, years	Histology at diagnosis	ER status	HER2 status	Pathological lymph node status	Tumor size, mm	Histological grade
1	61	IDC	N	P	N	31	3
2	60	IDC	P	N	P	29	2
3	48	IDC	P	P	N	30	1

*IDC* Invasive Ductal Carcinoma, *N* negative, *P* positive

### Tissue collection

Biopsies from normal and tumor tissue were taken in situ immediately after induction of anesthesia (by *intra-venous* injection of fentanyl and propofol) prior to radical mastectomy. All tumors in this study were well defined and easily palpable. Biopsies from normal tissue were taken as far from the tumor as possible. After surgical excision 4 biopsies were taken ex vivo, 3 from normal (to test for variation among replicate samples) and 1 from tumor tissue. Origin (tumor or normal) of the biopsies was confirmed by macroscopic inspection. All biopsies were snap frozen in liquid nitrogen and subsequently kept at − 80 °C until RNA extraction.

### RNA isolation and expression profiling

Total RNA was extracted using RNeasy Micro kit (Qiagen, Hilden, Germany) according to manufacturer’s instructions. RNA concentration and purity was determined by UV spectrometry on a NanoDrop 2000 (Thermo Scientific, Waltham, Massachusetts, USA). Amplified RNA was synthesized from 300 ng total RNA using the MessageAmpTM III RNA amplification kit and fragmented according to manufacturer’s instructions (Ambion, Austin, TX, USA). Fragmented amplified RNA was hybridized to Human Genome U133 Plus 2.0 GeneChip® (Affymetrix, Santa Clara, California, USA), washed and scanned as recommended by the manufacturer. Data are available from Gene Expression Omnibus (http://www.ncbi.nlm.nih.gov/geo; accession no. GSE71053).

### Data analysis and statistics

The affy package (www.bioconductor.org), implemented in the statistical programming language R version 3.1.1 was applied for initial data analysis. Robust multi-array average expression measure (rma) (Irizarry et al., 2003) was applied to perform background correction, quantile normalization, and expression index calculation of all microarrays. Only perfect match probes were used for data analysis.

Hierarchical clustering of genes was carried out using MultiExperiment Viewer (MeV; http://www.tm4.org) with Pearson correlation as distance metric and average linkage.

The experimental design is factorial with 3 putative effects on gene expression investigated simultaneously by GEE analysis. GEE was chosen because it allows analysis of interaction. The following analyses were performed. 1) Effect of ischemia during surgery on tumor tissue compared to normal (interaction). 2) Effect of surgical manipulation irrespective of tissue type/malignancy state (time). 3) Effect of malignancy state comparing tumor to normal tissue irrespective of whether samples had been collected pre or post surgery (tissue). A false discovery rate (FDR) of 0.01 was used for cut-off to identify differentially expressed genes. Subsequently, GSEA analysis was carried out to identify pathways differentially expressed in the same 3 comparisons using the GEE ranked data and the preranking option in GSEA. The GSEA method takes the entire rank of genes into account allowing identification of pathways not only represented among most differentially regulated genes, but also pathways supported by a larger number of moderately differentially expressed genes. The Reactome pathway collection from MSigDB was used for all GSEA analyses. For the ease of presentation of data a more stringent FDR cut-off of 0.005 was emploid for the GSEA analyses. Out of 674 gene sets, 486 passed a criterion of at least 15 genes represented in the data set.

In order to compare our results with a previous publication investigating the effect of cold ischemia on gene expression in tumor tissue, GSEA analysis was used to compare tumor tissue before and after surgical manipulation. Genes were in this analysis ranked according to differentially expression in tumor post-surgery compared to pre-surgery, using the statistical parameter *d*, derived from Significance Analysis of Microarray (SAM). Pathways with a FDR value below 0.0005 were considered significant.

## Results

**Fig. 1:**
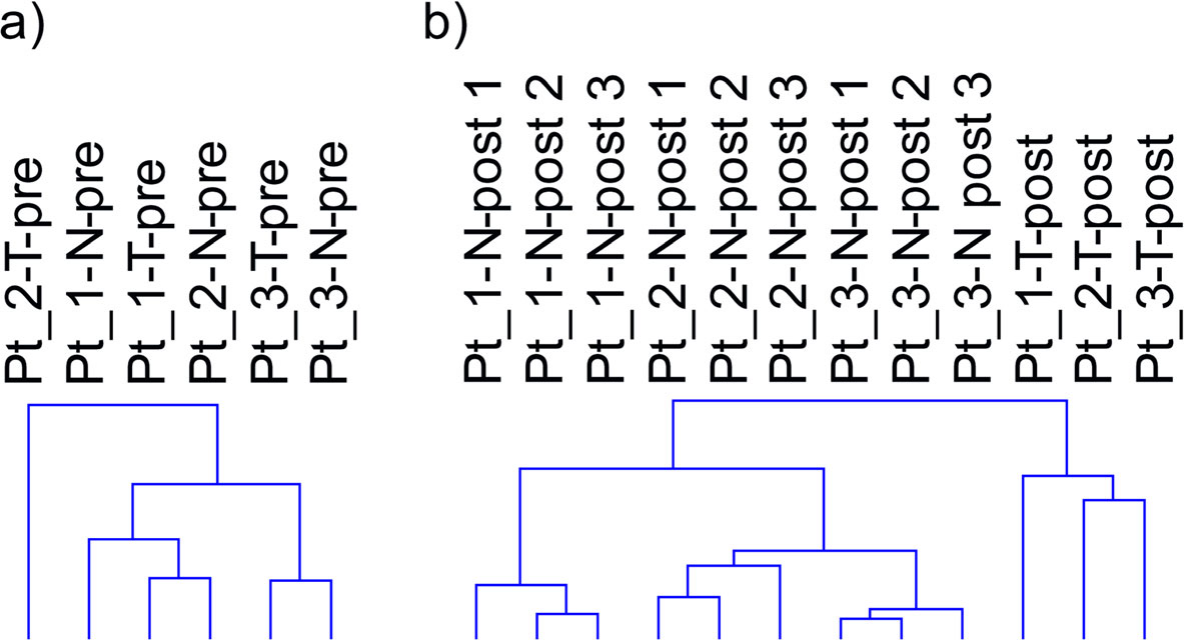
Hierarchical clustering dendrograms. Dendrograms based on hierarchical clustering of all genes on pre (**a**) and post (**b**) surgery samples. Patient samples are numbered Pt_1, Pt_2 and Pt_3, N for normal, T for tumor and pre/post for pre surgery and post-surgery respectively

**Fig. 2:**
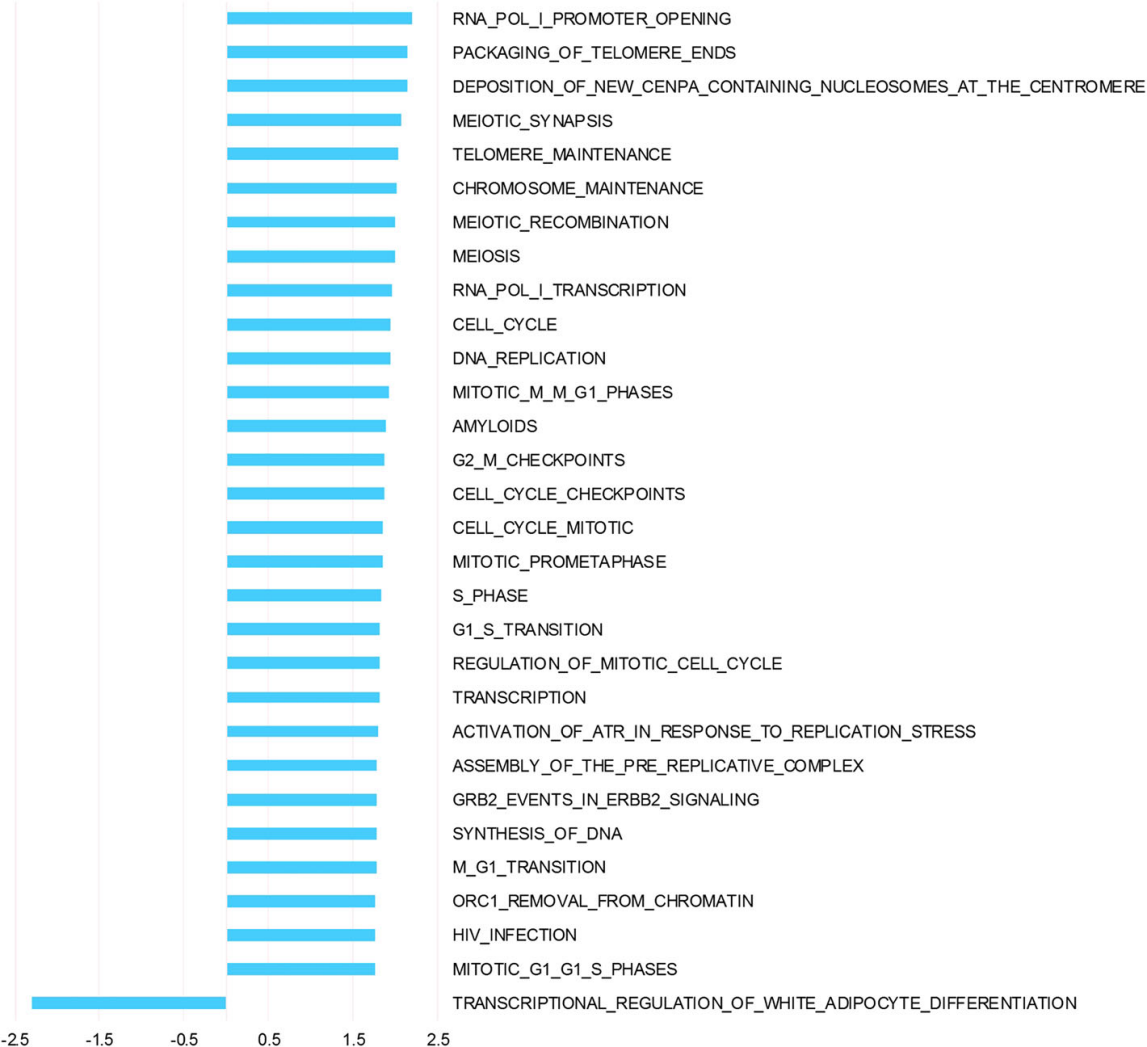
Pathway analysis of interaction. Pathway analysis was performed with GSEA using ranking of genes according to the interaction term from GEE. The Normalized Enrichment Scores (NES) is depicted for all Reactome pathways significantly altered in tumor tissue as a result of surgical manipulation compared to surgical manipulation of normal tissue

Hierarchical clustering of pre surgery samples using all genes (Fig 1a) shows a combined effect of individual and tissue type, with no distinct clustering of tumor and normal. Post surgery there is a more pronounced effect of tissue origin with tumor samples clustering separately from normal samples (Fig 1b). The 3 biopsies taken from normal tissue post surgery cluster together for each patient, as expected for tissue of the same tissue type handled identically. In order to analyze the interaction of ischemia (pre/post-surgery) and tissue (tumor/normal) GEE was applied. This analysis of the interaction identified 3179 differentially expressed genes. Top 50 up- and down-regulated genes are listed in additional material (Additional file 1 and Additional file 2). Pathway analysis of interaction, including all genes ranked by GEE and performed with GSEA, revealed significant up-regulation of 29 pathways and down-regulation of 1 pathway (Fig 2). Among the pathways specifically upregulated in tumor as a consequence of surgical manipulation were several cell cycle related pathways. Enrichment plots and information on pathways significantly affected by interaction are provided as additional material (Additional file 3 and Additional file 4).

**Fig. 3:**
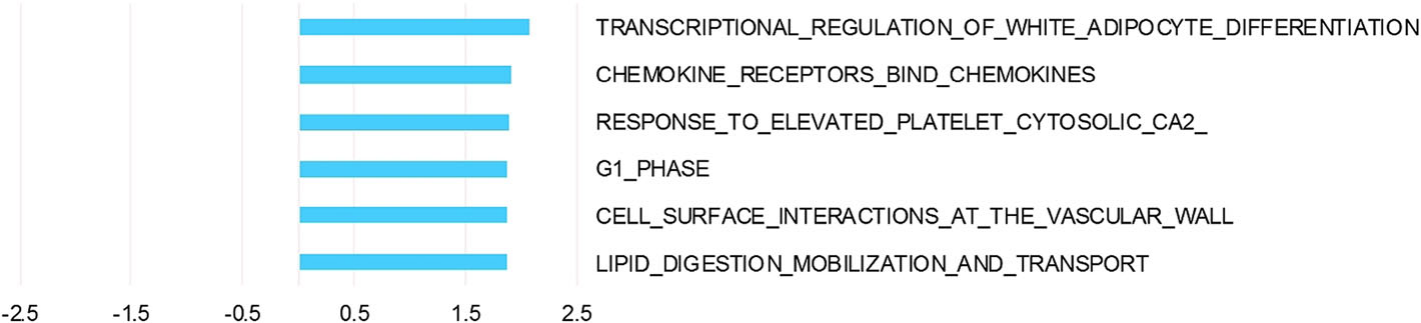
Pathway analysis of the effect of surgical manipulation (warm ischemia). Pathway analysis was performed with GSEA using ranking of genes according to the time term from GEE. NES depicted for all Reactome pathways significantly altered in samples collected post-surgery compared to samples collected pre-surgery irrespective of whether it was normal or tumor tissue

The analysis of ischemia, i.e. surgical manipulation, on tissue regardless of malignancy state identified 667 differentially expressed genes Top 50 up- and down-regulated genes are listed in additional material (Additional file 5 and Additional file 6). GSEA demonstrated 6 pathways upregulated after ischemia, including pathways involved in adipocyte differentiation, lipid mobilization, cell interactions and immune system pathways (Fig 3). Enrichment plots and information on pathways significantly affected by surgical manipulation are provided as additional material (Additional file 7 and Additional file 8).

**Fig. 4:**
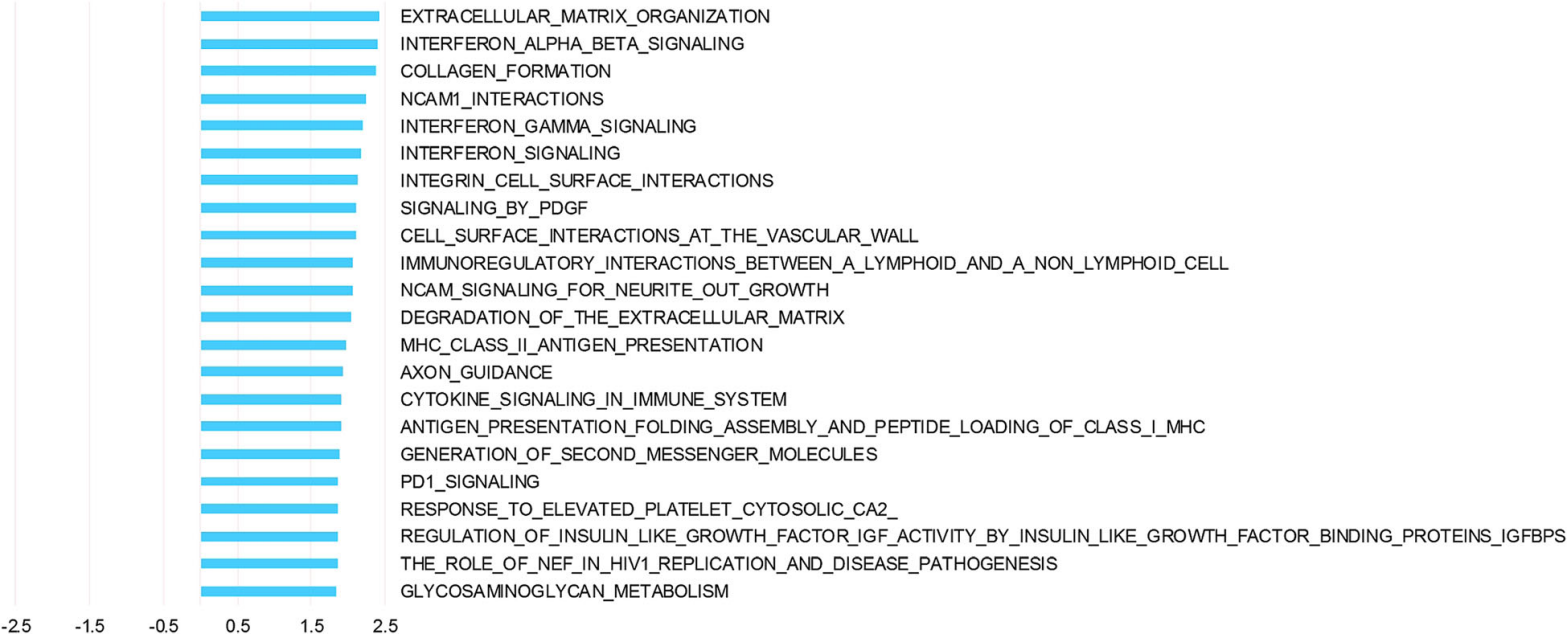
Pathway analysis of the effect of tissue type. Pathway analysis was performed with GSEA using ranking of genes according to the tissue term from GEE. NES depicted for all Reactome pathways significantly altered in tumor samples compared to normal samples irrespective of whether samples were collected pre- or post-surgery

The comparison of tumor tissue (pre- and post-surgery) and normal tissue (pre- and post-surgery) identified a total of 6166 differentially expressed genes. Top 50 up- and down-regulated genes are listed in additional material (Additional file 9 and Additional file 10). Pathway analysis demonstrated 22 pathways that were upregulated in tumor tissue compared to normal tissue (Fig 4). Among these pathways are pathways characteristic for invasive tumors such as extracellular matrix degradation, PDGF signaling and immune system pathways. Enrichment plots and information on pathways significantly affected by tissue type are provided as additional material (Additional file 11 and Additional file 12).

**Fig. 5:**
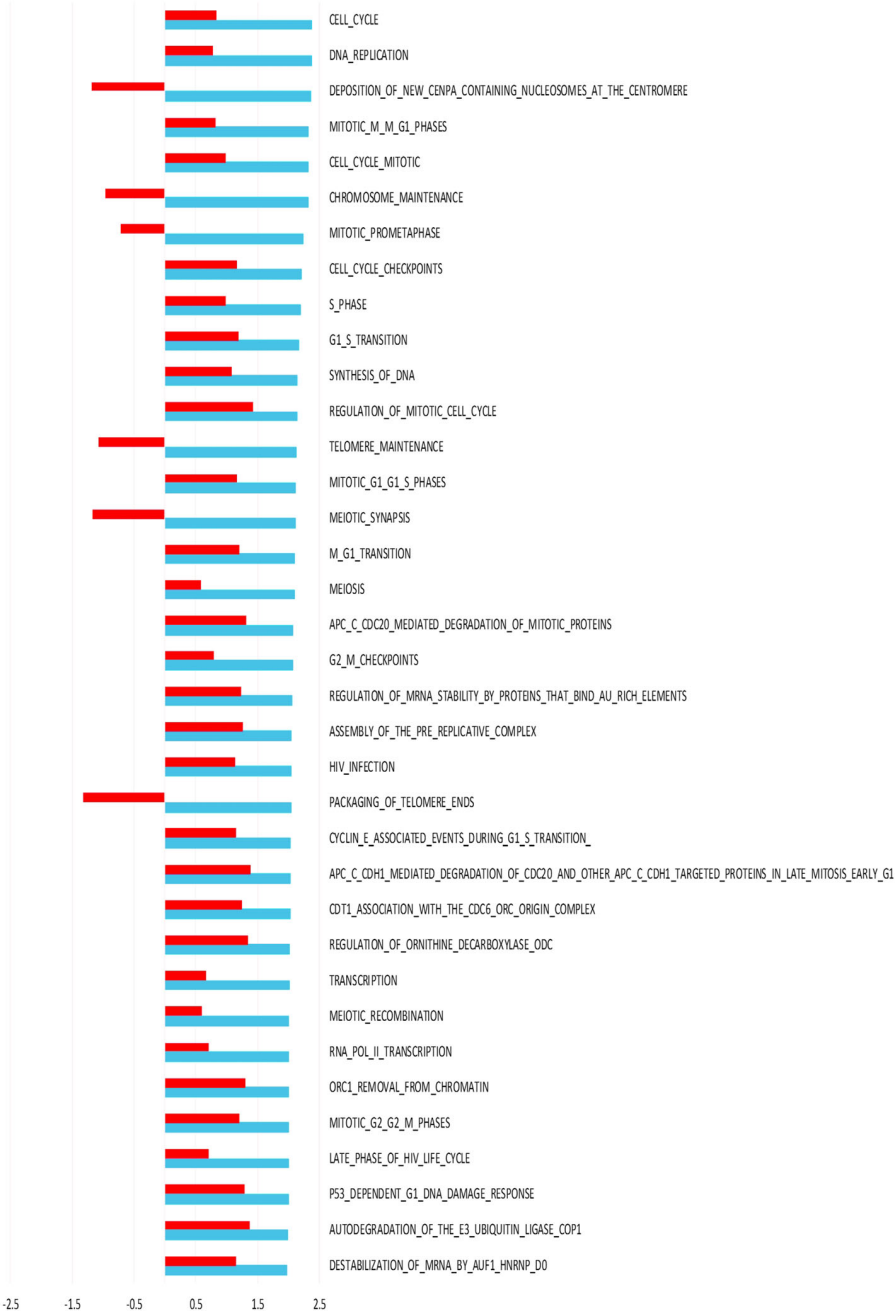
Comparison of GSEA on effect of warm and cold ischemia on tumor tissue. NES depicted in blue for all Reactome pathways significantly altered in tumor tissue collected post-surgery compared to pre-surgery (warm ischemia). To illustrate concordance with previously published data by Aktas et al. (2014) NES from tumor tissue subjected to 40 min cold ischemia post surgery are represented by red bars. One pathway had a nominal *p*-value < 0.05 in the dataset from Aktas: REGULATION_OF_MITOTIC_CELL_CYCLE

We next wanted to perform validation of our findings in external data sets. Since no data of warm ischemia are available, we used the data set from Aktas et al.’s study of cold eschemia in tumor tissue (Aktas et al., 2014) . This provided a strong validation of observed effect in tumor tissue (Fig 5) with 30 of 36 pathways being affected in the same direction, and 1 of these pathways (REGULATION_OF_MITOTIC_CELL_CYCLE) had a nominal *p*-value of less than 0.05.

## Discussion

Our study reveals a remarkable effect of surgical manipulation, such as warm ischemia and anesthesia. It is not possible from the current study to deduce the specific contribution of these variables. In our hierarchical clustering analysis it is only for the post-surgery samples that tumor samples cluster separately from normal samples, indicating a differential effect of surgical manipulation on gene expression profiles in normal and tumor tissue. Indeed GSEA analysis identifies several pathways which are differentially affected by warm ischemia between tumor and normal tissue. This is in keeping with observations of cell type specific response to environmental stress such as hypoxia (Chi et al., 2006) and a likely consequence of different pre-conditioning as a result of the different milieu of tumor cells compared to normal. Interestingly, immune system pathways are upregulated both as a result of surgical manipulation and when comparing tumor and normal tissue. This is in agreement with what has already been documented in the literature. (Grigoryev et al., 2006; Bottai et al., 2017; Liu et al., 2017). However, immune system pathways are not significantly affected in the interaction analysis, indicating activation of a similar immune response in tumor and normal tissue as a consequence of surgical manipulation.

Pathways preferentially enriched in tumor compared to normal tissue as a result of surgical manipulation are pathways involved in cell cycling. Despite it being well documented for neurons to re-enter cell cycling in response to ischemia (Love, 2003), this is a somewhat counterintuitive response for actively replicating cells. However, when restricting analysis to the effect of surgical manipulation on tumor tissue, 1 of the cell-cycle related pathways up regulated in tumor tissue as a result of surgery was found to be significantly up regulated as a result of 40 min cold ischemia of breast tumor tissue post surgery in a previous study by Aktas et al. (Aktas et al., 2014). Furthermore, there is a significant correlation between direction of the effect of warm and cold ischemia, with 30 out of 36 pathways affected in the same direction in both studies, providing strong validation of the observed effect on cell cycle pathways of ischemia on tumor tissue. Differences between this study and previous studies could be explained both by a differential effect of cold and warm ischemia and differences in baseline, since up regulation of different genes have been shown to peak at different time points after tissue resection (Spruessel et al., 2004). In previous studies baseline has been within minutes of surgery, effectively the endpoint of the current study. For the purpose of the current study it was of utmost importance to ensure immediate preservation of baseline samples. In other studies samples were pathologically evaluated before mincing into smaller fragments and dividing into aliquots for freezing at different time points (Aktas et al., 2014; Hatzis et al., 2010). This procedure minimizes intra-tumoral variation. However, it introduces a median delay prior to stabilization of 40 min, and hence would not be suitable in our set up. On the other hand, lack of control over tissue composition adds some degree of uncertainty to our results. It is also important to keep in mind that the three tumors in this study represent three different histopathological entities (Table 1). This could potentially affect the analysis and may explain the lack of clustering of tumor samples pre-surgery. Interestingly, our results suggest a distinct response of this heterogeneous group to surgical manipulation.

In order to elucidate differences in gene expression between tumor and normal tissue in addition to what is evident from hierarchical clustering, GSEA was carried out and 22 pathways displayed differential representation. This shows a difference between normal and tumor samples when it comes to pathways involved in organization and degradation of extracellular matrix and interferon signaling.

Speculatively, it may not be surprising that the differences between normal and tumor tissue in vivo are less pronounced than the differences after being subjected to ischemic stress. After all, the cells have to carry out more or less the same functions in order to survive. Pronounced difference in reaction to changes in external environment could putatively be what constitutes the main difference between normal cells and tumor cells. Activation of cell cycling genes during stress could be an acquired response of a cancer cell no longer reacting as expected to environmental signals. Similar to what is seen in cell cultures where normal cells stop growing when reaching confluence whereas tumor cells keep dividing.

Reported differential expression of pathways between tumor and normal tissue almost exclusively refers to post surgery samples. This has been proven to work well for several multigene classifiers. Our results open the possibility that differences observed in post-surgical specimens not necessarily mirrors differences present in vivo, and whereas difference in response to stress may be an equally good measurement for classifying tumors into subgroups, it requires standardized control over sampling conditions and information about surgical procedures. Furthermore, when it comes to investigating biological differences of tumor and normal cells and considering possible therapeutical targets, caution should be taken deducing in vivo differences based on gene expression profiles from tissue sampled after surgery.

## Conclusions

The current study is to our knowledge the first study to simultaneously investigate the effect of surgical manipulation, malignancy state and the interaction of these two parameters on gene expression profiling in breast tissue. The self-matched design gives added statistical power compared to a case-control design, and even with a limited number of samples we are able demonstrate an effect on a large number of pathways and a differential response between tumor and normal tissue. The majority of pathways affected primarily in tumor tissue are pathways that have previously been implicated in oncogenesis. It is of great interest that the well documented up regulation of gene sets involved in cell cycling may in fact primarily reflect altered reaction to surgical manipulation/ischemic stress of tumor cells compared to normal cells rather than reflecting the state of cells in the body.

## Additional files

Additional file 1: Top 50 up-regulated genes (interaction). The top 50 genes up-regulated in the GEE interaction analysis. (PDF 30 kb)

Additional file 2: Top 50 down-regulated genes (interaction). The top 50 genes down-regulated in the GEE interaction analysis. (PDF 31 kb)

Additional file 3: GSEA enrichment plots – interaction. GSEA enrichment plots of pathways significantly affected by interaction. (TIF 3730 kb)

Additional file 4: Significantly affected pathways – interaction. Information on rank and enrichment scores of significantly affected pathways in the GSEA analysis on interaction. (PDF 27 kb)

Additional file 5: Top 50 up-regulated genes (surgical manipulation). The top 50 genes up-regulated in the GEE surgical manipulation analysis. (PDF 31 kb)

Additional file 6: Top 50 down-regulated genes (surgical manipulation). The top 50 genes down-regulated in the GEE surgical manipulation analysis. (PDF 30 kb)

Additional file 7: GSEA enrichment plots – surgical manipulation. GSEA enrichment plots of pathways significantly affected by surgical manipulation. (TIF 1173 kb)

Additional file 8: Significantly affected pathways - surgical manipulation. Information on rank and enrichment scores of significantly affected pathways in the GSEA analysis on surgical manipulation. (PDF 9 kb)

Additional file 9: Top 50 up-regulated genes (tissue type). The top 50 genes up-regulated in the GEE tissue type analysis. (PDF 35 kb)

Additional file 10: Top 50 down-regulated genes (tissue type). The top 50 genes down-regulated in the GEE tissue type analysis. (PDF 35 kb)

Additional file 11: GSEA enrichment plots – tissue type. GSEA enrichment plots of pathways significantly affected by tissue type. (TIF 3588 kb)

Additional file 12: Significantly affected pathways - tissue type. Information on rank and enrichment scores of significantly affected pathways in the GSEA analysis on tissue type. (PDF 21 kb)

## Abbreviations

FDR: False Discovery Rate
GEE: Generalized Estimating Equation
GSEA: Gene Set Enrichment Analysis
MeV: MultiExperiment Viewer
rma: Robust multi-array average expression measure
SAM: Significance Analysis of Microarray
