# Supplementary material for: Differential effect of surgical manipulation on gene expression in normal breast tissue and breast tumor tissue
Source: Mol Med. 2018 Nov 16;24:57. doi: 10.1186/s10020-018-0058-x (PMC6240321; doi:10.1186/s10020-018-0058-x)
Supplement: Supplementary file 1 — Top 50 up-regulated genes (interaction). The top 50 genes up-regulated in the GEE interaction analysis. (PDF 30 kb) [file 10020_2018_58_MOESM1_ESM.pdf]

Upregulated genes (Interaction)

ID	Gene symbol	regression coefficient (interaction)	Fold Change (Interaction)	p(interaction)	q(interaction)
217718_s_at	YWHAB	0.51556496	1.429553844	1.32E-50	1.45E-46
221528_s_at	ELMO2	0.49688949	1.411167744	8.37E-45	5.09E-41
212007_at	UBXN4	0.532917498	1.446852143	3.43E-42	1.44E-38
235577_at	ZNF652	1.284166166	2.435412525	8.16E-38	2.79E-34
223680_at	ZNF607	0.790601382	1.729795371	8.30E-37	2.39E-33
203350_at	AP1G1	0.448424952	1.36454971	1.30E-35	3.56E-32
236491_at	BCL2L10	0.147842952	1.107911737	6.58E-34	1.71E-30
218140_x_at	SRPRB	0.406617326	1.325574106	5.09E-33	1.21E-29
1561135_at	---	0.421046633	1.338898534	2.16E-29	4.08E-26
230534_at	ZNF678	0.384866318	1.30573879	1.75E-28	3.09E-25
201697_s_at	DNMT1	0.773803866	1.709771897	2.79E-28	4.49E-25
1558611_at	CRHR1-IT1_///_MGC57346	0.241818784	1.18248246	1.25E-27	1.87E-24
222466_s_at	MRPL42	0.889701438	1.852792653	3.96E-24	3.94E-21
201999_s_at	DYNLT1	0.809023531	1.752025207	4.85E-24	4.73E-21
1560071_a_at	RP1-30M3.5	0.583817982	1.498810492	9.80E-24	9.40E-21
1562235_s_at	---	2.195987575	4.582032131	1.59E-23	1.50E-20
213516_at	---	0.107118888	1.077075131	7.76E-22	6.06E-19
206547_s_at	PPEF1	1.109624274	2.157894412	1.78E-21	1.35E-18
216644_at	---	0.509583426	1.423639065	1.96E-21	1.47E-18
1570567_at	---	0.399266619	1.318837321	4.75E-21	3.46E-18
202314_at	CYP51A1_///_LRRD1	1.085436737	2.122017769	6.16E-21	4.43E-18
223506_at	ZC3H8	0.594891138	1.510358614	7.10E-21	4.98E-18
236879_at	---	1.027824124	2.038946787	2.55E-20	1.68E-17
207986_x_at	CYB561	0.274952435	1.209954197	8.80E-20	5.41E-17
217677_at	PLEKHA2	0.100565821	1.072193892	1.54E-19	9.14E-17
235804_at	---	0.379230361	1.30064781	1.56E-19	9.14E-17
218470_at	YARS2	0.688529784	1.611640297	7.57E-19	4.18E-16
213781_at	PPP1R37	0.463848864	1.379216429	7.70E-19	4.21E-16
214079_at	DHRS2	5.859682219	58.06843384	1.19E-18	6.44E-16
1570130_at	SPATS2	0.240111812	1.181084194	3.21E-18	1.69E-15
233273_at	RP11-506O24.2	1.375727445	2.594987241	4.83E-18	2.47E-15
224990_at	SMIM14	1.319285267	2.495424521	8.91E-18	4.31E-15
217485_x_at	PMS2P1	0.272058417	1.207529486	1.03E-17	4.87E-15
216129_at	ATP9A	0.913385901	1.883460662	1.41E-17	6.65E-15
227943_at	RP11-196G18.24	1.899797993	3.731609427	2.74E-17	1.26E-14
201111_at	CSE1L	0.887495064	1.849961265	2.87E-17	1.31E-14
202299_s_at	LAMTOR5	0.439858086	1.356470889	3.43E-17	1.55E-14
237032_x_at	SIPA1L1	0.242676392	1.183185594	4.23E-17	1.90E-14
219429_at	FA2H	0.949298683	1.930933772	7.21E-17	3.18E-14
1555146_at	ATF2	0.261901967	1.199058433	9.41E-17	4.12E-14
212105_s_at	DHX9	0.67533247	1.596964752	1.49E-16	6.40E-14
243634_at	SMIM14	1.327997884	2.5105403	1.56E-16	6.66E-14
202882_x_at	NOL7	0.277269918	1.211899377	3.85E-16	1.56E-13
218340_s_at	UBA6	1.307828129	2.475685632	5.08E-16	2.03E-13
214373_at	---	0.518707635	1.432671288	7.80E-16	3.05E-13
228733_at	PUSL1	0.454419263	1.370231126	7.92E-16	3.07E-13
241920_x_at	SPG11	0.340498763	1.266194262	8.82E-16	3.37E-13
217428_s_at	COL10A1	5.617931386	49.10953939	1.28E-15	4.78E-13
216819_at	HSD3BP1_///_HSD3BP1	0.121201483	1.087640277	3.32E-15	1.20E-12
