# Supplementary material for: Differential effect of surgical manipulation on gene expression in normal breast tissue and breast tumor tissue
Source: Mol Med. 2018 Nov 16;24:57. doi: 10.1186/s10020-018-0058-x (PMC6240321; doi:10.1186/s10020-018-0058-x)
Supplement: Supplementary file 2 — Top 50 down-regulated genes (interaction). The top 50 genes down-regulated in the GEE interaction analysis. (PDF 31 kb) [file 10020_2018_58_MOESM2_ESM.pdf]

Downregulated genes

ID	Gene symbol	regression coefficient (interaction)	Fold Change (interaction)	p(interaction)	q(interaction)
210607_at	FLT3LG	-0.659993838	0.632881	2.03E-28	3.47E-25
224038_at	TIMM23	-0.126375935	0.91612989	2.38E-28	3.94E-25
1556533_at	LINC00868	-0.480661135	0.716649134	4.81E-28	7.51E-25
1564212_at	LOC101929762	-0.282981102	0.821890953	1.29E-27	1.87E-24
229524_at	EFCAB14	-0.262963611	0.833374227	1.30E-27	1.87E-24
1568647_at	LOC100505851	-0.217489787	0.860060595	1.95E-27	2.73E-24
243535_at	---	-0.221301457	0.857791273	3.46E-27	4.72E-24
217688_at	---	-0.277442627	0.825052239	3.54E-27	4.72E-24
237956_s_at	C5orf58	-0.330570595	0.795221906	4.40E-27	5.72E-24
236482_at	---	-0.406757846	0.754316638	5.13E-27	6.53E-24
228611_s_at	---	-0.512637165	0.700939987	7.69E-27	9.56E-24
1556874_a_at	MEX3C	-0.273996735	0.82702524	1.96E-26	2.39E-23
233629_at	---	-0.247089928	0.842594306	7.77E-26	9.23E-23
240734_at	LOC100507221	-0.286034799	0.820153128	8.56E-26	9.96E-23
210709_at	ZC3H15	-0.356216481	0.781210648	1.02E-25	1.16E-22
1562924_at	LOC340357	-0.240189192	0.846634279	3.39E-25	3.78E-22
212089_at	LMNA	-0.656167105	0.634561938	3.55E-25	3.88E-22
1570239_a_at	CLNK	-0.301665969	0.81131498	9.14E-25	9.80E-22
236899_at	---	-0.158894899	0.89571092	1.70E-24	1.78E-21
216865_at	COL14A1	-0.488628725	0.712702196	3.11E-24	3.21E-21
1561737_at	---	-0.199102008	0.871092598	3.50E-24	3.54E-21
1556771_a_at	CNTFR-AS1	-0.353427981	0.782722064	1.97E-23	1.82E-20
222482_at	SSBP3	-0.343834245	0.78794441	5.32E-23	4.85E-20
234484_s_at	ACSS1	-0.328436043	0.796399354	6.51E-23	5.83E-20
240742_at	---	-0.32040665	0.800844113	7.65E-23	6.75E-20
216068_at	---	-0.055789872	0.962067569	9.53E-23	8.27E-20
209286_at	CDC42EP3	-1.503985768	0.352577968	1.08E-22	9.20E-20
242375_x_at	LOC101929036_///_PAH	-0.12158118	0.919179686	1.11E-22	9.33E-20
1562601_at	LINC01121	-0.256792208	0.83694678	2.27E-22	1.88E-19
216271_x_at	SYDE1	-0.218173799	0.859652919	6.57E-22	5.33E-19
234130_at	LDB3	-0.386571475	0.764945317	6.63E-22	5.33E-19
204222_s_at	GLIPR1	-0.942115165	0.52046925	6.75E-22	5.35E-19
219170_at	FSD1	-0.489293543	0.712373847	1.54E-21	1.19E-18
1553261_x_at	ALS2CR11	-0.176911371	0.884594777	4.16E-21	3.07E-18
230977_at	NPM2	-0.166439128	0.89103924	7.05E-21	4.98E-18
227262_at	HAPLN3	-0.349566279	0.784820005	1.18E-20	8.20E-18
227565_at	KLHL5	-1.044411103	0.484842777	2.12E-20	1.45E-17
207004_at	BCL2	-0.372465752	0.772461132	2.15E-20	1.45E-17
225685_at	CDC42EP3	-1.582330761	0.333941949	2.22E-20	1.48E-17
241204_at	---	-0.361319474	0.778452289	3.83E-20	2.50E-17
210706_s_at	RNF24	-0.198673487	0.871351375	5.21E-20	3.35E-17
228778_at	MCPH1	-0.471567298	0.721180704	6.42E-20	4.08E-17
214371_at	TSSK2	-0.274629419	0.826662633	6.72E-20	4.18E-17
32094_at	CHST3	-0.492660413	0.710713292	6.72E-20	4.18E-17
1565713_at	---	-0.171877096	0.887686957	1.17E-19	7.11E-17
213386_at	TMEM246	-0.429514141	0.7425118	1.46E-19	8.79E-17
202150_s_at	NEDD9	-0.743449938	0.597309288	1.93E-19	1.12E-16
217088_s_at	NCR1	-0.276863763	0.825383348	2.12E-19	1.22E-16
229108_at	---	-0.332977725	0.793896188	3.39E-19	1.93E-16
234034_at	---	-0.266464717	0.831354262	4.93E-19	2.78E-16
