# Supplementary figures and images for: Differential effect of surgical manipulation on gene expression in normal breast tissue and breast tumor tissue
Source: Mol Med. 2018 Nov 16;24:57. doi: 10.1186/s10020-018-0058-x (PMC6240321; doi:10.1186/s10020-018-0058-x)

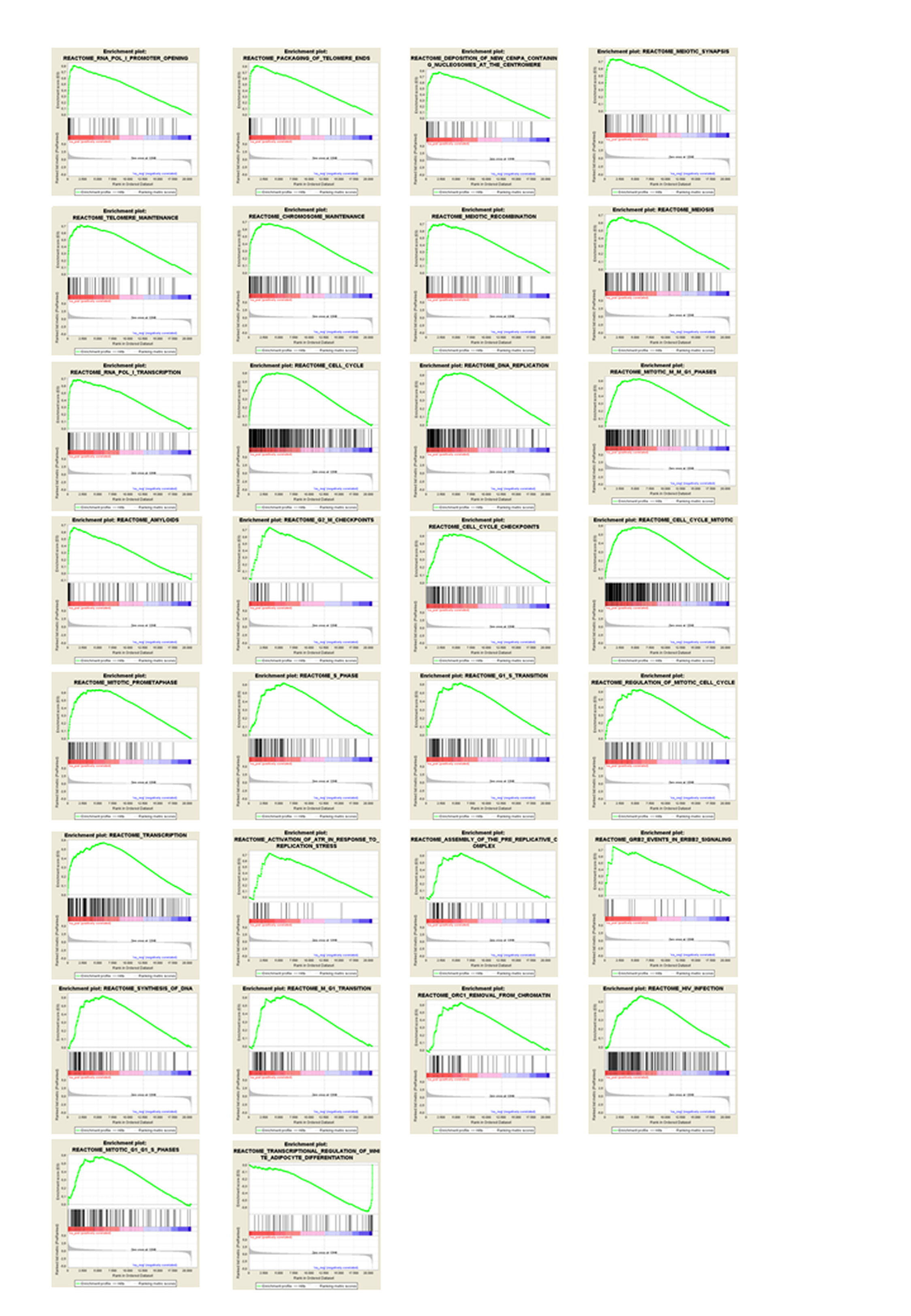

Supplement: Supplementary file 3 — GSEA enrichment plots – interaction. GSEA enrichment plots of pathways significantly affected by interaction. (TIF 3730 kb) [file 10020_2018_58_MOESM3_ESM.tif]

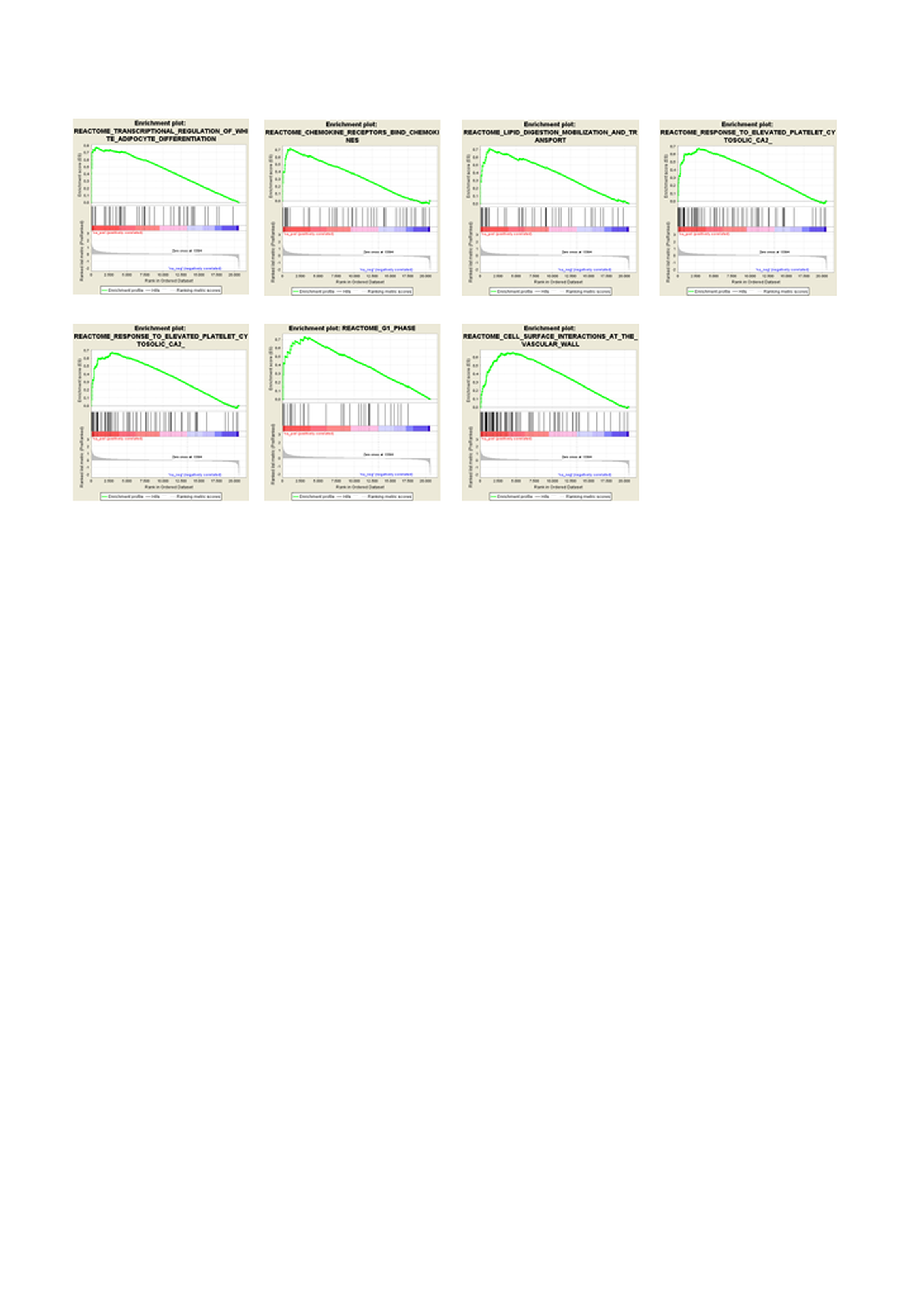

Supplement: Supplementary file 7 — GSEA enrichment plots – surgical manipulation. GSEA enrichment plots of pathways significantly affected by surgical manipulation. (TIF 1173 kb) [file 10020_2018_58_MOESM7_ESM.tif]

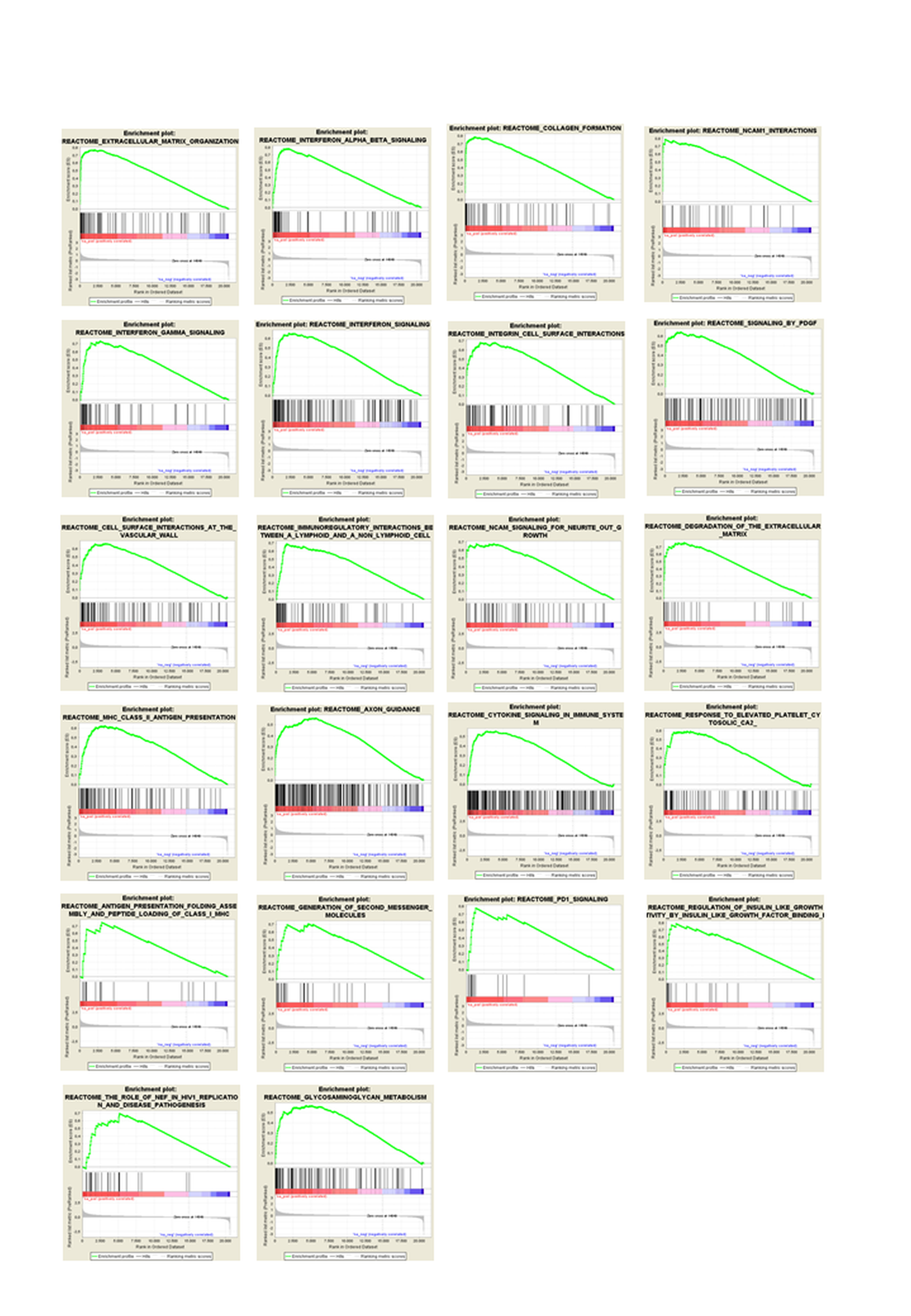

Supplement: Supplementary file 11 — GSEA enrichment plots – tissue type. GSEA enrichment plots of pathways significantly affected by tissue type. (TIF 3588 kb) [file 10020_2018_58_MOESM11_ESM.tif]
