# Supplementary material for: Differential effect of surgical manipulation on gene expression in normal breast tissue and breast tumor tissue
Source: Mol Med. 2018 Nov 16;24:57. doi: 10.1186/s10020-018-0058-x (PMC6240321; doi:10.1186/s10020-018-0058-x)
Supplement: Supplementary file 4 — Significantly affected pathways – interaction. Information on rank and enrichment scores of significantly affected pathways in the GSEA analysis on interaction. (PDF 27 kb) [file 10020_2018_58_MOESM4_ESM.pdf]

NAME	SIZE	ES	NES	NOM p-val	FDR q-val	FWER p-val	RANK AT MAX	LEADING EDGE
REACTOME_RNA_POL_I_PROMOTER_OPENING	47	0.8114803	2.1871815	0	0	0	988	tags=38%, list=5%, signal=40%
REACTOME_PACKAGING_OF_TELOMERE_ENDS	39	0.81921226	2.1465638	0	0	0	1290	tags=44%, list=6%, signal=46%
REACTOME_DEPOSITION_OF_NEW_CENPA_CONTAINING_NUCLEOSOMES_AT_THE_CENTROMERE	50	0.7841518	2.1417966	0	0	0	2092	tags=52%, list=10%, signal=58%
REACTOME_MEIOTIC_SYNAPSIS	60	0.7404236	2.068315	0	0	0	1290	tags=33%, list=6%, signal=35%
REACTOME_TELOMERE_MAINTENANCE	62	0.71815425	2.0262043	0	1.93E-04	0.001	2099	tags=40%, list=10%, signal=45%
REACTOME_CHROMOSOME_MAINTENANCE	100	0.67798203	2.0219612	0	1.61E-04	0.001	2099	tags=38%, list=10%, signal=42%
REACTOME_MEIOTIC_RECOMBINATION	70	0.7068457	1.9992437	0	1.38E-04	0.001	2792	tags=37%, list=14%, signal=43%
REACTOME_MEIOSIS	96	0.6751805	1.998243	0	1.21E-04	0.001	2792	tags=34%, list=14%, signal=40%
REACTOME_RNA_POL_I_TRANSCRIPTION	70	0.6914593	1.968253	0	3.16E-04	0.003	1571	tags=30%, list=8%, signal=32%
REACTOME_CELL_CYCLE	345	0.60343045	1.9443841	0	3.80E-04	0.004	4863	tags=51%, list=24%, signal=66%
REACTOME_DNA_REPLICATION	166	0.6271394	1.936099	0	3.46E-04	0.004	5666	tags=64%, list=27%, signal=88%
REACTOME_MITOTIC_M_M_G1_PHASES	147	0.6298976	1.9259815	0	4.75E-04	0.006	5648	tags=65%, list=27%, signal=88%
REACTOME_AMYLOIDS	66	0.67019045	1.8850827	0	0.001018795	0.014	988	tags=27%, list=5%, signal=29%
REACTOME_G2_M_CHECKPOINTS	32	0.739597	1.8681419	0.001234568	0.001420209	0.021	3362	tags=66%, list=16%, signal=78%
REACTOME_CELL_CYCLE_CHECKPOINTS	100	0.62692964	1.8639838	0	0.001452816	0.023	4453	tags=56%, list=22%, signal=71%
REACTOME_CELL_CYCLE_MITOTIC	271	0.58339554	1.8549417	0	0.001423541	0.024	4863	tags=52%, list=24%, signal=67%
REACTOME_MITOTIC_PROMETAPHASE	74	0.6412991	1.8528863	0	0.001339803	0.024	5169	tags=57%, list=25%, signal=75%
REACTOME_S_PHASE	95	0.62072074	1.8350911	0	0.001789641	0.034	5775	tags=68%, list=28%, signal=95%
REACTOME_G1_S_TRANSITION	94	0.61842316	1.8207523	0	0.00214606	0.041	5648	tags=65%, list=27%, signal=89%
REACTOME_REGULATION_OF_MITOTIC_CELL_CYCLE	73	0.62734926	1.8110863	0	0.002565568	0.05	5775	tags=68%, list=28%, signal=95%
REACTOME_TRANSCRIPTION	178	0.5734964	1.8053831	0	0.002624965	0.054	5941	tags=54%, list=29%, signal=76%
REACTOME_ACTIVATION_OF_ATR_IN_RESPONSE_TO_REPLICATION_STRESS	27	0.73008776	1.7971648	0	0.00306404	0.067	3362	tags=67%, list=16%, signal=80%
REACTOME_ASSEMBLY_OF_THE_PRE_REPLICATIVE_COMPLEX	56	0.6375512	1.7810187	0	0.00371228	0.085	5775	tags=75%, list=28%, signal=104%
REACTOME_GRB2_EVENTS_IN_ERBB2_SIGNALING	21	0.7517525	1.7784184	0.001278772	0.003795798	0.091	1330	tags=38%, list=6%, signal=41%
REACTOME_SYNTHESIS_OF_DNA	80	0.6211803	1.7765441	0	0.003943081	0.099	5775	tags=70%, list=28%, signal=97%
REACTOME_M_G1_TRANSITION	69	0.62019247	1.7710038	0	0.004156463	0.107	5775	tags=71%, list=28%, signal=98%
REACTOME_ORC1_REMOVAL_FROM_CHROMATIN	58	0.63258404	1.766709	0.001154734	0.004561747	0.122	5775	tags=72%, list=28%, signal=100%
REACTOME_HIV_INFECTION	189	0.56319004	1.758982	0	0.004975662	0.138	5981	tags=57%, list=29%, signal=80%
REACTOME_MITOTIC_G1_G1_S_PHASES	115	0.58119786	1.7585679	0	0.004869551	0.14	4463	tags=51%, list=22%, signal=65%
REACTOME_TRANSCRIPTIONAL_REGULATION_OF_WHITE_ADIPOCYTE_DIFFERENTIATION	53	-0.66070986	-2.2949464	0	0	0	693	tags=21%, list=3%, signal=21%
