# Supplementary material for: Differential effect of surgical manipulation on gene expression in normal breast tissue and breast tumor tissue
Source: Mol Med. 2018 Nov 16;24:57. doi: 10.1186/s10020-018-0058-x (PMC6240321; doi:10.1186/s10020-018-0058-x)
Supplement: Supplementary file 5 — Top 50 up-regulated genes (surgical manipulation). The top 50 genes up-regulated in the GEE surgical manipulation analysis. (PDF 31 kb) [file 10020_2018_58_MOESM5_ESM.pdf]

Upregulated genes (time)

ID	Gene symbol	regression coefficient(time)	Fold Change(time)	p(time)	q(time)
241041_at	---	0.211357735	1.157777268	1.27E-58	3.48E-54
201464_x_at	JUN	1.303769987	2.468731585	8.25E-55	1.13E-50
206115_at	EGR3	2.242112739	4.730893681	2.58E-54	2.82E-50
239777_at	C14orf182	0.240084248	1.181061629	1.62E-36	1.27E-32
240199_x_at	ZNF345	0.09212875	1.06594186	3.53E-36	2.41E-32
244621_x_at	---	0.144041063	1.104995935	8.50E-33	5.17E-29
237923_at	---	0.081143704	1.057856329	1.18E-29	5.84E-26
1554210_at	ZCCHC13	0.198810181	1.147751391	3.64E-29	1.57E-25
244485_at	HLA-DPB1	0.361952825	1.285164313	3.74E-29	1.57E-25
201289_at	CYR61	3.009463914	8.052651586	3.36E-27	1.31E-23
212614_at	ARID5B	0.507909442	1.42198815	2.44E-25	7.84E-22
202672_s_at	ATF3	2.507248151	5.685345999	1.07E-24	3.26E-21
240133_x_at	---	0.098529328	1.070681462	1.23E-24	3.54E-21
210764_s_at	CYR61	2.856702579	7.243578401	6.21E-24	1.62E-20
239365_at	---	0.114851268	1.082863404	3.65E-23	9.08E-20
1558577_at	LOC148709	0.227185347	1.170549016	5.09E-23	1.16E-19
240082_s_at	C17orf74	0.139153791	1.101258986	4.24E-21	9.26E-18
208339_at	XKRY_///_XKRY2	0.119451196	1.086321545	2.04E-19	3.99E-16
227744_s_at	HNRNPD	0.165446458	1.121513089	2.51E-18	4.43E-15
231578_at	GBP1	0.457083834	1.372764199	1.30E-15	1.73E-12
1557890_at	STXBP5-AS1	0.098900326	1.07095683	6.57E-15	7.98E-12
219228_at	ZNF331	0.504878077	1.419003431	1.17E-14	1.39E-11
215466_at	---	0.20153523	1.149921382	1.47E-14	1.71E-11
208239_at	FOXE1	0.289719749	1.222402797	1.84E-14	2.05E-11
1555939_at	LOC101928001	0.12781883	1.09264052	2.43E-14	2.66E-11
243716_at	---	0.146456307	1.10684738	2.53E-14	2.72E-11
1562290_at	---	0.097617415	1.070004908	5.01E-14	5.16E-11
1561593_at	BC032916_///_RP11-400D2.2	0.093324234	1.066825514	5.09E-14	5.16E-11
213281_at	JUN	1.270480716	2.412419356	2.51E-13	2.36E-10
208020_s_at	CACNA1C	0.149257559	1.108998612	3.63E-13	3.30E-10
217666_at	---	0.125709378	1.091044071	5.24E-13	4.62E-10
240647_at	---	0.127452375	1.092363016	6.65E-13	5.77E-10
243259_at	ATXN7	0.320401546	1.248678046	7.93E-13	6.77E-10
244493_at	GPR22	0.18676279	1.138206868	4.78E-12	3.79E-09
200623_s_at	CALM1_///_CALM2_///_CALM3	0.258646717	1.196355967	7.04E-12	5.50E-09
209774_x_at	CXCL2	2.225378995	4.676337305	1.09E-11	8.30E-09
1556996_at	LEPROT	0.228090062	1.171283298	1.28E-11	9.34E-09
202791_s_at	PPP6R2	0.038506902	1.027050344	1.70E-11	1.21E-08
217476_at	NR1D1_///_THRA	0.29900547	1.23029601	2.38E-11	1.62E-08
201466_s_at	JUN	1.810593493	3.507865648	3.49E-11	2.36E-08
1558999_x_at	LOC283922_///_PDPR	0.08590416	1.061352699	3.95E-11	2.60E-08
243678_at	---	0.088846	1.063519142	4.16E-11	2.71E-08
203922_s_at	CYBB	0.599796866	1.515503166	4.54E-11	2.92E-08
211646_at	IGH_///_IGHA1_///_IGHG1_///_IGHG2_///_IGHG3_///_IGHM_///_LOC102725526	0.154182824	1.112791129	6.15E-11	3.91E-08
201693_s_at	EGR1	2.198594471	4.590319175	7.59E-11	4.77E-08
1559362_at	---	0.304529657	1.235015935	7.79E-11	4.84E-08
227613_at	ZNF331	0.717466418	1.644291882	1.15E-10	6.99E-08
241587_at	NAALADL2-AS3	0.176743509	1.130329592	1.20E-10	7.19E-08
223594_at	TMEM117	0.265878519	1.202368	1.30E-10	7.71E-08
233018_at	TMEM134	0.064237163	1.04553196	1.57E-10	9.21E-08
