# Supplementary material for: Differential effect of surgical manipulation on gene expression in normal breast tissue and breast tumor tissue
Source: Mol Med. 2018 Nov 16;24:57. doi: 10.1186/s10020-018-0058-x (PMC6240321; doi:10.1186/s10020-018-0058-x)
Supplement: Supplementary file 6 — Top 50 down-regulated genes (surgical manipulation). The top 50 genes down-regulated in the GEE surgical manipulation analysis. (PDF 30 kb) [file 10020_2018_58_MOESM6_ESM.pdf]

Downregulated genes (time)

ID	Gene symbol	regression coefficient(time)	Fold Change(time)	p(time)	q(time)
239243_at	ZNF638_///_ZNF638-IT1	-0.88586512	0.541162914	9.44E-60	5.16E-55
1554841_at	MTHFD2L	-0.319796131	0.801183086	5.23E-56	9.53E-52
243974_at	---	-0.481155215	0.716403745	1.58E-37	1.44E-33
217482_at	---	-0.485814387	0.714093857	2.81E-32	1.54E-28
228801_at	ORMDL1	-0.214294127	0.861967795	1.62E-26	5.90E-23
242906_at	SESN3	-0.224015521	0.856179073	4.18E-26	1.43E-22
1564204_at	LINC00869	-0.185496688	0.879346285	4.33E-24	1.18E-20
231136_at	---	-0.645663094	0.639198933	4.22E-23	1.00E-19
239649_at	---	-0.659085195	0.633279729	8.16E-21	1.72E-17
240859_at	ZFYVE16	-0.49389904	0.710103371	1.93E-19	3.90E-16
1568856_at	NBR1	-0.144709263	0.90456165	6.93E-19	1.31E-15
239285_at	---	-0.27567773	0.82606217	8.97E-19	1.64E-15
232668_at	---	-0.361437221	0.778388758	4.41E-18	7.53E-15
214107_x_at	LOC440434	-0.262846474	0.833441894	4.72E-18	7.82E-15
230058_at	SDCCAG3	-0.346173669	0.786667742	1.14E-17	1.83E-14
215597_x_at	---	-0.287460668	0.819342941	7.61E-17	1.19E-13
237369_at	---	-0.520132389	0.697307842	2.95E-16	4.48E-13
236961_at	---	-0.48094582	0.716507733	4.30E-16	6.35E-13
1558578_a_at	C7orf73_///_LOC101930655_///_SLC13A4	-0.141942448	0.906298092	4.73E-16	6.80E-13
228122_at	CCDC66	-0.303653455	0.810198065	6.33E-16	8.87E-13
241343_at	RNASEH1	-0.148122018	0.9024244	8.06E-16	1.10E-12
231332_at	---	-0.423981447	0.745364779	3.49E-15	4.55E-12
218337_at	FAM160B2	-0.23269604	0.851043016	4.01E-15	5.10E-12
230712_at	LOC101930059_///_LOC102724250_///_NBPF1_///_NBPF11_///_NBPF15	-0.429824001	0.742352342	4.64E-15	5.77E-12
214861_at	KDM4C	-0.542881976	0.686398366	1.82E-14	2.05E-11
1558467_a_at	UGGT2	-0.474541362	0.719695547	3.68E-14	3.87E-11
242337_at	---	-0.250295031	0.840724469	8.53E-14	8.47E-11
239811_at	---	-0.425517764	0.744571467	1.37E-13	1.33E-10
232565_at	---	-0.24647029	0.842956278	1.72E-13	1.65E-10
242901_at	---	-0.384701903	0.765937244	3.34E-13	3.10E-10
235217_at	LINC01004	-0.589263782	0.664682013	4.78E-13	4.28E-10
238214_at	LRRC69	-0.319218016	0.801504199	1.75E-12	1.47E-09
236007_at	AKAP10	-0.475184184	0.719374944	2.53E-12	2.10E-09
220969_s_at	---	-0.426021855	0.744311352	3.28E-12	2.67E-09
225659_at	SPOPL	-0.230062771	0.852597795	3.51E-12	2.82E-09
242553_at	ABCC3	-0.269716813	0.829482349	8.64E-12	6.65E-09
1568857_a_at	NBR1	-0.544454614	0.685650552	1.12E-11	8.36E-09
244001_at	NAP1L4	-0.136374785	0.909802444	1.15E-11	8.49E-09
230099_at	---	-0.359518232	0.779424814	1.54E-11	1.11E-08
1568864_at	LOC100131691	-0.141323414	0.906687051	1.72E-11	1.21E-08
210720_s_at	NECAB3	-0.285098401	0.820685631	2.11E-11	1.46E-08
243751_at	CHD2	-0.362019845	0.778074474	3.91E-11	2.60E-08
235613_at	---	-0.413049923	0.751033974	1.15E-10	6.99E-08
212786_at	CLEC16A	-0.131680712	0.912767474	1.93E-10	1.12E-07
234164_at	---	-0.29860793	0.813036525	2.11E-10	1.21E-07
222057_at	NOL12	-0.203395024	0.868504348	3.24E-10	1.84E-07
242980_at	COMMD10	-0.166118674	0.891237181	3.96E-10	2.21E-07
242637_at	---	-0.31342139	0.804731053	6.71E-10	3.63E-07
213499_at	CLCN2	-0.131745031	0.912726781	7.17E-10	3.84E-07
