# Supplementary material for: Differential effect of surgical manipulation on gene expression in normal breast tissue and breast tumor tissue
Source: Mol Med. 2018 Nov 16;24:57. doi: 10.1186/s10020-018-0058-x (PMC6240321; doi:10.1186/s10020-018-0058-x)
Supplement: Supplementary file 8 — Significantly affected pathways - surgical manipulation. Information on rank and enrichment scores of significantly affected pathways in the GSEA analysis on surgical manipulation. (PDF 9 kb) [file 10020_2018_58_MOESM8_ESM.pdf]

NAME	SIZE	ES	NES	NOM p-val	FDR q-val	FWER p-val	RANK AT MAX	LEADING EDGE
REACTOME_TRANSCRIPTIONAL_REGULATION_OF_WHITE_ADIPOCYTE_DIFFERENTIATION	53	0.7737551	2.0834239	0	0	0	616	tags=25%, list=3%, signal=25%
REACTOME_CHEMOKINE_RECEPTORS_BIND_CHEMOKINES	48	0.7174357	1.9083016	0	0.004608898	0.009	1008	tags=27%, list=5%, signal=28%
REACTOME_RESPONSE_TO_ELEVATED_PLATELET_CYTOSOLIC_CA2_	75	0.6701494	1.9035074	0	0.003413945	0.01	2913	tags=43%, list=14%, signal=50%
REACTOME_G1_PHASE	34	0.7270455	1.8838503	0	0.002818954	0.011	3021	tags=38%, list=15%, signal=45%
REACTOME_CELL_SURFACE_INTERACTIONS_AT_THE_VASCULAR_WALL	84	0.65308154	1.8825482	0	0.002460464	0.012	4524	tags=60%, list=22%, signal=76%
REACTOME_LIPID_DIGESTION_MOBILIZATION_AND_TRANSPORT	43	0.7129337	1.880336	0	0.002217446	0.013	1244	tags=28%, list=6%, signal=30%
