# Supplementary material for: Differential effect of surgical manipulation on gene expression in normal breast tissue and breast tumor tissue
Source: Mol Med. 2018 Nov 16;24:57. doi: 10.1186/s10020-018-0058-x (PMC6240321; doi:10.1186/s10020-018-0058-x)
Supplement: Supplementary file 9 — Top 50 up-regulated genes (tissue type). The top 50 genes up-regulated in the GEE tissue type analysis. (PDF 35 kb) [file 10020_2018_58_MOESM9_ESM.pdf]

Upregulated genes (tissue)

ID	Gene symbol	regression coefficient(time)	regression coefficient(tissue)	Fold Change (tissue)	p(tissue)	q(tissue)
215633_x_at	LST1	0.299175556	0.544294175	1.458306707	0	0
1555603_at	BAGE	0.182663	0.147012406	1.107274106	0	0
220614_s_at	ADGB	0.183851381	0.214088616	1.159970902	0	0
206305_s_at	C8A	0.10555943	0.107844333	1.077616864	0	0
1561136_at	GYPE	0.083164941	0.181908958	1.134383897	0	0
213942_at	MEGF6	-0.069583095	0.16736721	1.123007225	0	0
211582_x_at	LST1	0.150349626	0.454235969	1.37005705	0	0
228112_at	DNAH1	0.158548842	0.301194544	1.232164216	0	0
1555638_a_at	SAMSN1	0.247919349	0.399875354	1.319393913	0	0
244754_at	---	-0.132669872	0.078186475	1.055690162	0	0
240493_at	---	-0.101634127	0.141997686	1.10343197	0	0
234159_at	---	-0.091361733	0.156266895	1.114399793	0	0
1552312_a_at	MFAP3	0.258124528	0.267167826	1.203443012	0	0
1562675_at	C1orf86	0.055658652	0.129096499	1.093608605	0	0
235951_s_at	ZNF688	0.072582903	0.244006608	1.184277036	0	0
223152_at	PPP1R12C	0.161855944	0.334529614	1.260966208	0	0
200945_s_at	SEC31A	0.102068567	0.308436607	1.238365004	0	0
229443_at	UQCC2	0.063336548	0.119315446	1.086219333	0	0
244739_at	RDX	0.041027025	0.063345837	1.044886209	0	0
1570320_at	---	0.025850092	0.110377014	1.079510304	0	0
205790_at	SKAP1	0.165641377	0.289234796	1.221991962	0	0
217610_at	SPDYE2	-0.066967291	0.151888503	1.111022861	0	0
214096_s_at	SHMT2	-0.102200057	0.3047501	1.235204659	0	0
219479_at	KDELC1	0.155653512	0.81614176	1.760691025	0	0
202329_at	CSK	0.058959467	0.575448312	1.490140447	0	0
206255_at	BLK	0.018623225	0.571352259	1.485915689	0	0
240290_at	RP11-646J21.6	0.016186686	0.076923199	1.054766166	0	0
213691_at	LOC101928378	0.0174986	0.190499162	1.14115848	0	0
208829_at	TAPBP	-0.076171467	0.530925903	1.444856188	0	0
230424_at	NREP	-0.041914379	0.336087435	1.262328534	0	0
1553817_at	LOC102725315_///_LOC727983_///_POM121L10P_///_POM121L1P_///_POM121L4P_///_POM121L8P_///_POM121L9P	0.018496076	0.051679069	1.03647051	0	0
201653_at	CNIH1	0.02585037	0.147225394	1.107437588	0	0
229003_x_at	FAM69B	0.00467163	0.187003145	1.13839651	0	0
209415_at	FZR1	-0.011409605	0.105942944	1.076197562	0	0
224421_x_at	PMCHL1	0.083844914	0.14691046	1.107195865	3.76E-306	4.03E-303
237221_at	---	0.165717178	0.179178369	1.132238879	1.36E-299	1.40E-296
236482_at	---	0.251140142	0.355768095	1.279666698	3.42E-282	3.40E-279
201063_at	RCN1	0.190344734	0.612251026	1.528642479	1.82E-281	1.77E-278
215967_s_at	LY9	0.03016747	0.113843396	1.082107176	2.34E-281	2.24E-278
240734_at	LOC100507221	0.152622298	0.195349302	1.145001352	1.66E-276	1.54E-273
240022_at	ZC3H4	0.071032394	0.174532638	1.128598735	1.75E-267	1.60E-264
208464_at	GRIA4	0.046152606	0.157764672	1.115557341	2.59E-258	2.24E-255
1557302_at	ZNF585B	0.014593929	0.120058229	1.086778725	1.58E-248	1.31E-245
233188_at	GALNT18	0.12072532	0.214494194	1.160297045	2.68E-248	2.19E-245
242009_at	SLC6A4	-0.005487089	0.176231791	1.12992874	3.37E-246	2.71E-243
227707_at	MYLIP	0.082222223	0.238720506	1.179945729	2.30E-238	1.82E-235
1558565_at	AKNA	0.097491605	0.151531638	1.110748072	4.74E-238	3.71E-235
203101_s_at	MGAT2	-0.050874301	0.176317244	1.129995669	3.56E-236	2.74E-233
214617_at	PRF1	0.22690858	0.377860968	1.299413833	1.01E-234	7.65E-232
239687_at	SLX4	-0.075150096	0.059551855	1.04214199	3.13E-233	2.35E-230
