# Supplementary material for: Differential effect of surgical manipulation on gene expression in normal breast tissue and breast tumor tissue
Source: Mol Med. 2018 Nov 16;24:57. doi: 10.1186/s10020-018-0058-x (PMC6240321; doi:10.1186/s10020-018-0058-x)
Supplement: Supplementary file 10 — Top 50 down-regulated genes (tissue type). The top 50 genes down-regulated in the GEE tissue type analysis. (PDF 35 kb) [file 10020_2018_58_MOESM10_ESM.pdf]

Downregulated genes (tissue)

ID	Gene symbol	regression coefficient(time)	regression c	Fold Change	p(tissue)	q(tissue)
229870_at	LOC644656	-0.693617473	-0.36419944	0.77689986	0	0
238146_at	---	-0.505031442	-0.81818745	0.56715405	0	0
238880_at	GTF3A	-0.46050377	-0.38160268	0.76758441	0	0
235862_at	---	-0.139172298	-0.19448469	0.87388498	0	0
200862_at	DHCR24	-0.636248443	-0.58776479	0.66537299	0	0
1566163_at	---	-0.354308673	-0.41769067	0.74862199	0	0
1555397_at	MYO1D	-0.164433416	-0.21640152	0.86070961	0	0
241728_at	---	-0.278335399	-0.54920801	0.68339519	0	0
1557360_at	LRPPRC	-0.37311013	-0.43630896	0.73902293	0	0
241395_at	NIT1	-0.221403119	-0.35286189	0.78302925	0	0
212845_at	SAMD4A	0.414417381	-0.39716373	0.75934966	0	0
230843_at	---	-0.137799561	-0.22676658	0.85454799	0	0
1569569_x_at	---	-0.11108082	-0.24778449	0.84218875	0	0
206658_at	UPK3B	-0.041220514	-0.07828205	0.94718487	0	0
206562_s_at	CSNK1A1	-0.114762091	-0.23679191	0.8486303	0	0
218997_at	POLR1E	0.064823931	-0.07618312	0.94856391	0	0
243378_at	---	-0.163477221	-0.09877687	0.93382436	6.71E-304	7.06E-301
227930_at	AGO4	-0.07584437	-0.31882645	0.80172177	8.09E-296	8.19E-293
230085_at	PDK3	-0.40327662	-0.79891555	0.57478107	3.64E-277	3.43E-274
233314_at	---	-0.343539431	-0.51624202	0.69919074	3.18E-265	2.85E-262
244674_at	---	-0.354117047	-0.6333349	0.64468445	2.36E-263	2.08E-260
201807_at	VPS26A	0.155482539	-0.23202748	0.85143749	8.58E-253	7.33E-250
203774_at	MTR	0.085200715	-0.20198001	0.8693566	1.16E-250	9.75E-248
226684_at	ATG2B	-0.06771103	-0.23801528	0.84791098	1.00E-232	7.42E-230
235225_at	SCN2B	-0.063977306	-0.17207154	0.88756732	1.42E-218	9.60E-216
216614_at	---	-0.094639934	-0.5517547	0.6821899	5.26E-218	3.50E-215
239079_at	ADAM7	-0.256108187	-0.19569806	0.87315031	2.94E-214	1.91E-211
222849_s_at	SCRN3	0.045713083	-0.29372896	0.81579074	1.92E-203	1.18E-200
201443_s_at	ATP6AP2	-0.066288313	-0.15687177	0.89696788	2.30E-203	1.40E-200
226938_at	DCAF4	-0.066718489	-0.04815804	0.96717037	5.85E-199	3.48E-196
207365_x_at	USP34	-0.279796353	-0.13751097	0.90908622	7.30E-198	4.29E-195
235435_at	AASDH	-0.114659675	-0.19109303	0.87594183	1.04E-192	5.92E-190
1554324_s_at	DYNC2LI1	-0.064875001	-0.18116097	0.88199295	2.53E-187	1.41E-184
214594_x_at	ATP8B1	-0.488567461	-0.47388804	0.72002154	1.45E-183	7.84E-181
1560433_at	---	-0.10461409	-0.36894104	0.77435068	3.36E-169	1.73E-166
237647_at	GHRL	-0.103155857	-0.10594683	0.92919492	5.28E-167	2.67E-164
243550_at	ZDHHC21	-0.172147938	-0.36911543	0.77425708	6.35E-165	3.16E-162
215528_at	---	-0.185440994	-0.33873281	0.79073555	4.76E-164	2.34E-161
1557813_at	---	-0.37009024	-0.3353424	0.792596	2.09E-159	1.01E-156
237136_at	---	0.065832407	-0.09679151	0.93511033	2.25E-159	1.08E-156
232932_at	---	-0.061998336	-0.40875515	0.75327307	2.34E-155	1.10E-152
1570143_at	---	-0.688228029	-0.33687494	0.7917545	5.81E-151	2.69E-148
1569956_at	MYLK	0.185479059	-0.54636715	0.68474221	8.34E-151	3.83E-148
212591_at	ARID4B_///_RBM34	0.186357916	-0.15157074	0.90026976	4.84E-149	2.20E-146
225187_at	CCAR2	-0.305286033	-0.18241435	0.88122703	1.99E-145	8.91E-143
229949_at	---	-0.429425212	-0.49392169	0.71009222	2.36E-144	1.05E-141
219242_at	CEP63	0.126928196	-0.33303156	0.79386656	4.17E-136	1.81E-133
207717_s_at	PKP2	-0.465935149	-0.93810174	0.52191916	5.03E-135	2.17E-132
219415_at	TTYH1	0.036585822	-0.28267209	0.82206701	4.76E-132	2.03E-129
237389_at	RP11-676J12.4	-0.091359673	-0.38998824	0.76313582	2.31E-127	9.78E-125
