# Supplementary material for: Differential effect of surgical manipulation on gene expression in normal breast tissue and breast tumor tissue
Source: Mol Med. 2018 Nov 16;24:57. doi: 10.1186/s10020-018-0058-x (PMC6240321; doi:10.1186/s10020-018-0058-x)
Supplement: Supplementary file 12 — Significantly affected pathways - tissue type. Information on rank and enrichment scores of significantly affected pathways in the GSEA analysis on tissue type. (PDF 21 kb) [file 10020_2018_58_MOESM12_ESM.pdf]

NAME	SIZE	ES	NES	NOM p-val	FDR q-val	FWER p-val	RANK AT MAX	LEADING EDGE
REACTOME_EXTRACELLULAR_MATRIX_ORGANIZATION	83	0.7703211	2.4281466	0	0	0	2007	tags=48%, list=10%, signal=53%
REACTOME_INTERFERON_ALPHA_BETA_SIGNALING	59	0.7832882	2.3931856	0	0	0	2215	tags=58%, list=11%, signal=64%
REACTOME_COLLAGEN_FORMATION	56	0.7862881	2.3882022	0	0	0	1370	tags=45%, list=7%, signal=48%
REACTOME_NCAM1_INTERACTIONS	37	0.78820205	2.2465281	0	0	0	333	tags=30%, list=2%, signal=30%
REACTOME_INTERFERON_GAMMA_SIGNALING	54	0.7337933	2.1916099	0	0	0	2759	tags=61%, list=13%, signal=70%
REACTOME_INTERFERON_SIGNALING	143	0.6545483	2.1774087	0	0	0	2215	tags=39%, list=11%, signal=44%
REACTOME_INTEGRIN_CELL_SURFACE_INTERACTIONS	78	0.6828052	2.128378	0	0	0	1900	tags=38%, list=9%, signal=42%
REACTOME_SIGNALING_BY_PDGF	111	0.6449647	2.1170275	0	0	0	2123	tags=27%, list=10%, signal=30%
REACTOME_CELL_SURFACE_INTERACTIONS_AT_THE_VASCULAR_WALL	84	0.6636481	2.1046784	0	0	0	3489	tags=48%, list=17%, signal=57%
REACTOME_IMMUNOREGULATORY_INTERACTIONS_BETWEEN_A_LYMPHOID_AND_A_NON_LYMPHOID_CELL	59	0.69054884	2.070178	0	0	0	1466	tags=42%, list=7%, signal=45%
REACTOME_NCAM_SIGNALING_FOR_NEURITE_OUT_GROWTH	61	0.67606145	2.0583484	0	8.92E-05	0.001	3848	tags=46%, list=19%, signal=56%
REACTOME_DEGRADATION_OF_THE_EXTRACELLULAR_MATRIX	27	0.7582804	2.0399354	0	1.64E-04	0.002	2826	tags=56%, list=14%, signal=64%
REACTOME_MHC_CLASS_II_ANTIGEN_PRESENTATION	82	0.6247649	1.9726732	0	2.26E-04	0.003	3576	tags=48%, list=17%, signal=57%
REACTOME_AXON_GUIDANCE	232	0.5597155	1.9279453	0	7.76E-04	0.011	5628	tags=53%, list=27%, signal=72%
REACTOME_CYTOKINE_SIGNALING_IN_IMMUNE_SYSTEM	245	0.55222416	1.9057128	0	9.20E-04	0.014	2788	tags=34%, list=14%, signal=39%
REACTOME_ANTIGEN_PRESENTATION_FOLDING_ASSEMBLY_AND_PEPTIDE_LOADING_OF_CLASS_I_MHC	18	0.75238216	1.9023689	0	8.63E-04	0.014	3041	tags=61%, list=15%, signal=72%
REACTOME_GENERATION_OF_SECOND_MESSENGER_MOLECULES	24	0.70853555	1.8786261	0	0.001453612	0.025	4461	tags=71%, list=22%, signal=90%
REACTOME_PD1_SIGNALING	15	0.777693	1.87199	0	0.001482137	0.027	1322	tags=60%, list=6%, signal=64%
REACTOME_RESPONSE_TO_ELEVATED_PLATELET_CYTOSOLIC_CA2_	75	0.5932403	1.8714603	0	0.001455167	0.028	3673	tags=41%, list=18%, signal=50%
REACTOME_REGULATION_OF_INSULIN_LIKE_GROWTH_FACTOR_IGF_ACTIVITY_BY_INSULIN_LIKE_GROWTH_FACTOR_BINDING_PROTEINS_IGFBPS	16	0.78617686	1.8646673	0	0.001631163	0.033	1369	tags=50%, list=7%, signal=54%
REACTOME_THE_ROLE_OF_NEF_IN_HIV1_REPLICATION_AND_DISEASE_PATHOGENESIS	27	0.6955891	1.8629823	0	0.001601338	0.034	5169	tags=81%, list=25%, signal=109%
REACTOME_GLYCOSAMINOGLYCAN_METABOLISM	93	0.56875753	1.8333312	0	0.003552901	0.076	5096	tags=48%, list=25%, signal=64%
